# Generation of *Hprt*-disrupted rat through mouse←rat ES chimeras

**DOI:** 10.1038/srep24215

**Published:** 2016-04-11

**Authors:** Ayako Isotani, Kazuo Yamagata, Masaru Okabe, Masahito Ikawa

**Affiliations:** 1 Immunology Frontier Research Center, Osaka University, Yamadaoka 3-1, Suita, Osaka 565-0871, Japan; 2 Research Institute for Microbial Diseases, Osaka University, Yamadaoka 3-1, Suita, Osaka 565-0871, Japan

## Abstract

We established rat embryonic stem (ES) cell lines from a double transgenic rat line which harbours *CAG-GFP* for ubiquitous expression of GFP in somatic cells and *Acr3-EGFP* for expression in sperm (green body and green sperm: GBGS rat). By injecting the GBGS rat ES cells into mouse blastocysts and transplanting them into pseudopregnant mice, rat spermatozoa were produced in mouse←rat ES chimeras. Rat spermatozoa from the chimeric testis were able to fertilize eggs by testicular sperm extraction combined with intracytoplasmic sperm injection (TESE-ICSI). In the present paper, we disrupted rat hypoxanthine-guanine phosphoribosyl transferase (*Hprt*) gene in ES cells and produced a *Hprt*-disrupted rat line using the mouse←rat ES chimera system. The mouse←rat ES chimera system demonstrated the dual advantages of space conservation and a clear indication of germ line transmission in knockout rat production.

The investigation of gene functions has intensified following completion of genome projects in many species. A significant number of gene disrupted mouse lines have been produced using ES cells and the homologous recombination technique, for example, helping clarify the basic biology while serving as animal models for human diseases^1^. As a result, mice have replaced rats as the most popularly used experimental animal.

However, rats retain advantages over mice as experimental animals^2^,^3^. Their bigger body size facilitates experimental operations and repeated collection of blood samples. Moreover, rats are better suited to behavioural studies as they learn tricks, such as pressing a lever to get alcohol, more easily than mice. Despite the potential demand for gene disruption in rats, the isolation of rat ES cells was unsuccessful for many years. In 2008, Buehr and Li independently used MEK and GSK3 inhibitors as key factors in establishing the first rat ES cells^4^,^5^. Even today, however, there are few reports on gene disrupted rats involving ES cells^6^,^7^,^8^,^9^,^10^. The reason is unclear, but we presume it is more challenging to produce knockout (KO) rat lines than mice lines. Firstly, rat embryos are difficult to handle *in vitro*^11^. Secondly, it seemed that rat ES cell karyotypes became unstable during passages *in vitro*^5^. Thirdly, rats require greater space in animal facilities. If the efficiency of germline transmission (GLT) for KO rat production is low, the need for more animals and further space may hinder continued experiments.

Previously, we reported a successful production of a mouse←rat ES chimera (here, the ‘←’ indicates the rat ES cells were injected into mouse blastocysts to make chimera using mice as recipient mothers) and a contribution of rat ES cells to germ line cells^12^. In this system, preparation of rat embryos for production of rat spermatozoa is unnecessary. Therefore, animal and space requirements were identical to those for the gene disruption experiment in the mice. This enabled an enhanced experimental scale, in case the efficiency of GLT of rat ES cells was low. Both mouse and rat spermatozoa were produced in the chimeric testes. However, the shape of the sperm heads differ in rats and mice, and the utilization of GFP facilitated selection of rat spermatozoa and subsequent microinsemination^13^,^14^.

With these strategies in mind, we chose hypoxanthine-guanine phosphoribosyl transferase (*Hprt*) as a target gene and sought to demonstrate the new method to produce a gene-disrupted rat line using the mouse←rat ES chimera system. *Hprt* was chosen because the Lesch-Nyhan syndrome (LNS) was known to be caused by *HPRT* gene mutation in human. However, when the *Hprt-*deficient mice were made, they failed to show characteristic nervous disorders exhibited in LNS. One purpose of this study was to examine whether *Hprt* gene disruption in rats evokes self-injurious behaviour (SIB), as rats are better suited to behavioural studies and some drugs induce SIB in rats but not in mice^15^.

## Results

### Establishment of GFP-tagged rat ES cell lines

**Figure 1:**
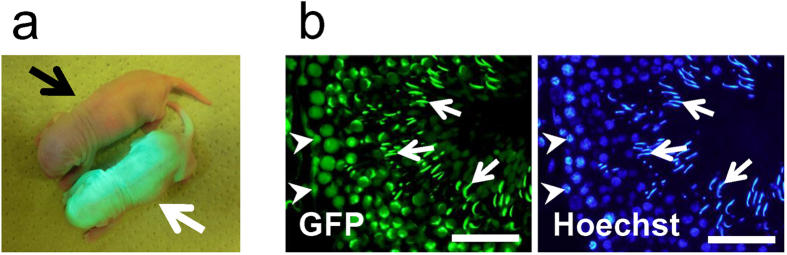
Characterizations of GBGS-rat. (**a**) F1 pups of GBGS-rat were obtained by the founder female GBGS-rat with male F344 rat of albino strain. The white arrow shows F1 GBGS-rat with GFP (rGBGS#6), while the black arrow shows wild-type rat without GFP. (**b**) Testis section of adult GBGS-rat (rGBGS#6). The GFP-signal was located in testicular somatic cells (arrowheads) and spermatogenic cells including sperm heads (arrows), shown in the left panel. The right panel shows Hoechst 33342 staining indicating nuclei. Scale bars represent 50 μm.

We used *CAG-EGFP*^16^ and the *Acr3-EGFP*^17^ to produce double transgenic F344 rat lines. All six established transgenic rat lines F344-Tg (CAG/Acr3-EGFP) which we abbreviated as GBGS (green body, green sperm) had ubiquitous fluorescence signal from somatic cells and testicular spermatozoa (Fig. 1 and Supplementary Fig. S1). We chose the rGBGS#6 rat line showing the strongest GFP signals for the establishment of the ES cell line.

**Figure 2:**
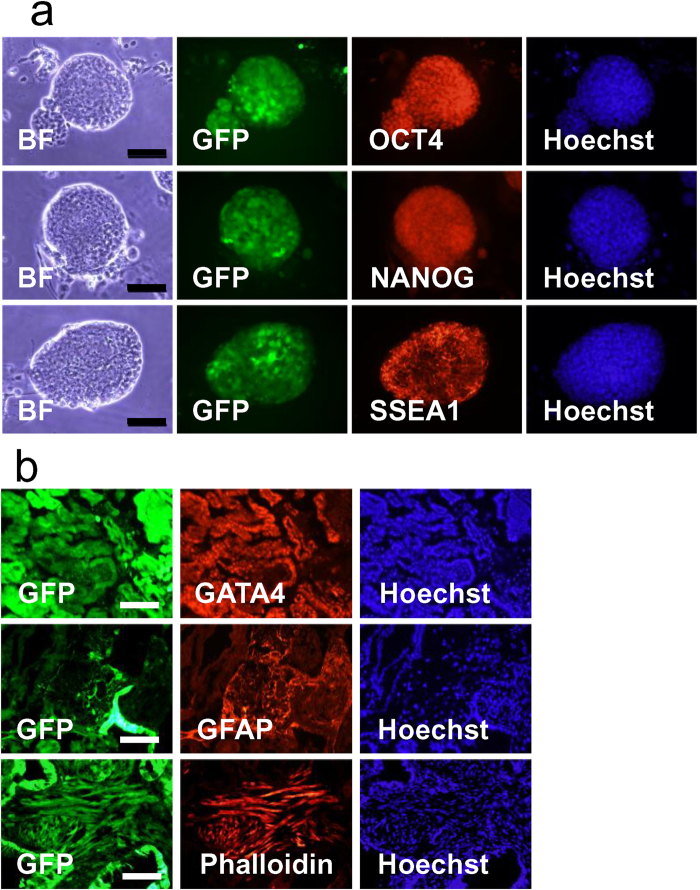
Expression of pluripotency markers in GBGS-rat ES cell line and teratoma formation. (**a**) A representative expressions of stem cell markers in the established GBGS rat ES cells (rGBGS-ES-104). Immunofluorescence staining for Oct 4 (sc-9081, Santa Cruz, 1:100), Nanog (ab80892, abcam, 1:100), SSEA1 (MC-480, R&D, 1:200). Scale bars represent 50 μm. (**b**) Immunohistochemistry of teratoma derived from GBGS rat ES cell line (rGBGS-ES-104). Approximately 10,000 cells were injected into testes of SCID mice. Teratomas were collected two months later. Histological sections of teratoma derived from GBGS-ES-104 show gastrointestinal-like epithelium (GATA4: sc-25310, Santa Cruz, 1:50), astrocyte (GFAP: #MAB360, MILLIPORE, 1:100), and skeletal muscle (Phalloidin: R415, Invitrogen, 1:250). GFP (green signal) indicates rat ES cells or rat ES cell-derived cells. Alexa Fluor-546 (red fluorescence) conjugated secondary antibodies (Invitrogen) were used at 1:500 dilutions, respectively. Hoechst 33342 (blue fluorescence) indicates nuclei. Scale bars represent 200 μm.

ES cell lines with a normal karyotype in over 65% of the cells were established from F344-GBGS inbred (three XY-type ES cell lines) and Wistar/F344-GBGS hybrid (three XY-type ES cell lines) (Supplementary Table S1) rats. The stemness was examined by the expression of Oct-4, Nanog and SSEA1 (Fig. 2a). Teratoma formations were observed after injecting the ES cell lines into the SCID mice testes. All of these established cell lines were shown to have the potential to differentiate into all three germ layers (Fig. 2b).

### Production of rat spermatozoa in the mouse←rat ES chimera

**Figure 3:**
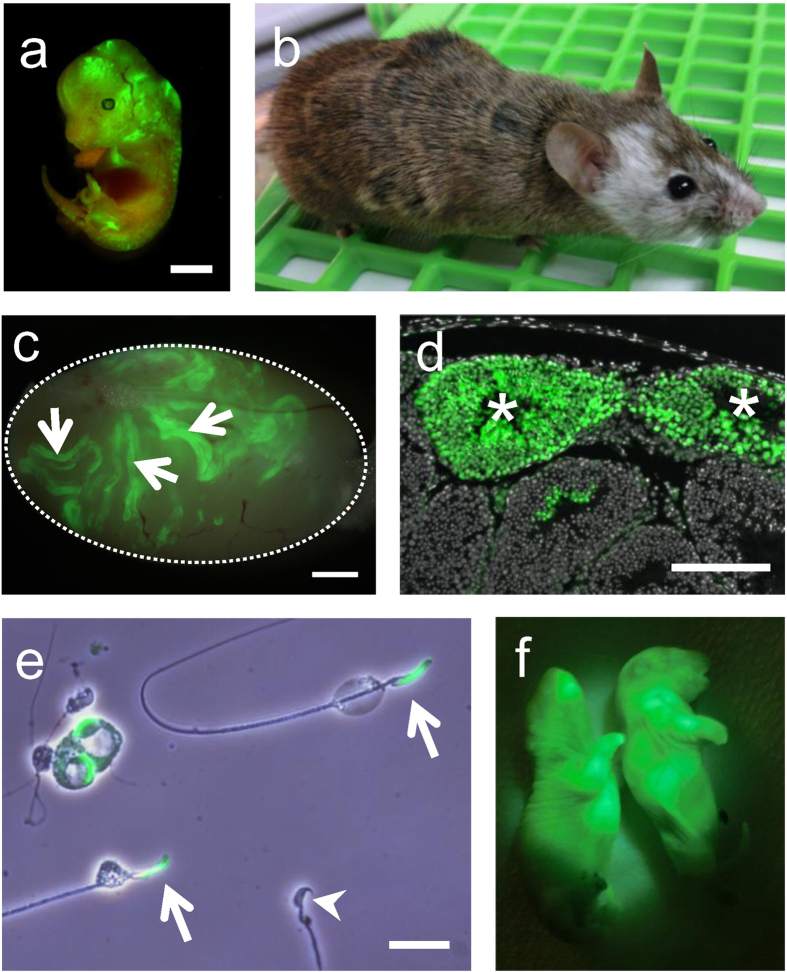
Production of mouse←rat ES chimera using GBGS-rat ES cell line and fertilizing rat spermatozoa. (**a**,**b**) The mouse←rat ES chimera using GBGS rat ES cells (rGBGS-ES-104). GFP signals indicate rat ES derived cells in E13.5 chimeric embryo (**a**). The white coat colour originated from rat ES cells and agouti coat colour derived from mice (**b**). (**c**) A testis from 10-week-old chimera. Rat derived cells are fluorescent green (arrows). (**d**) The testicular section of 10- week-old mouse←rat ES chimera. Progressions of spermatogenesis from rat spermatogonia were evident from the expression of GFP (asterisks). Cell nuclei were counterstained with Hoechst 33342 (gray). (**e**) Testicular spermatozoa from 10-week-old mouse←rat ES chimeric testis. Arrows indicate rat spermatozoa with GFP in sperm acrosome, while the arrowhead indicates a non-green mouse spermatozoon. (**f**) Rat pups derived from the rat spermatozoa in the mouse←rat ES chimeric testis. Scale bars show 1 mm (**a**,**c**), 200 μm (**d**), 20 μm (**e**).

To evaluate the competency for GLT in chimeric testes, we injected six lines of rat ES cells into E3.5 mouse blastocysts and nine chimeric males were obtained from each ES cell line (Fig. 3a,b and Supplementary Table S1). None of the ES cell lines derived from F344-GBGS contributed to germ cells (Supplementary Table S1). However, two out of the three ES cell lines from Wistar/F344-GBGS contributed in testicular germ cells and rat spermatozoa were formed in chimeric testes (Fig. 3c–e, Supplementary Table S1).

We chose one of the Wistar/F344-GBGS ES cell lines (rGBGS-ES-104) for the following experiments, as this cell line showed the highest normal karyotype rate and the most efficient GLT potency which was retained even at 16 passages (Supplementary Table S1). In the next experiment, we examined if such rat spermatozoa from the chimeric testis could fertilize eggs by TESE-ICSI^13^,^14^. As a result, two offspring were obtained from 93 TESE-ICSI fertilized eggs (Fig. 3f and Supplementary Table S2).

### Production of *Hprt*-disrupted rat using the mouse←rat ES chimera

**Figure 4:**
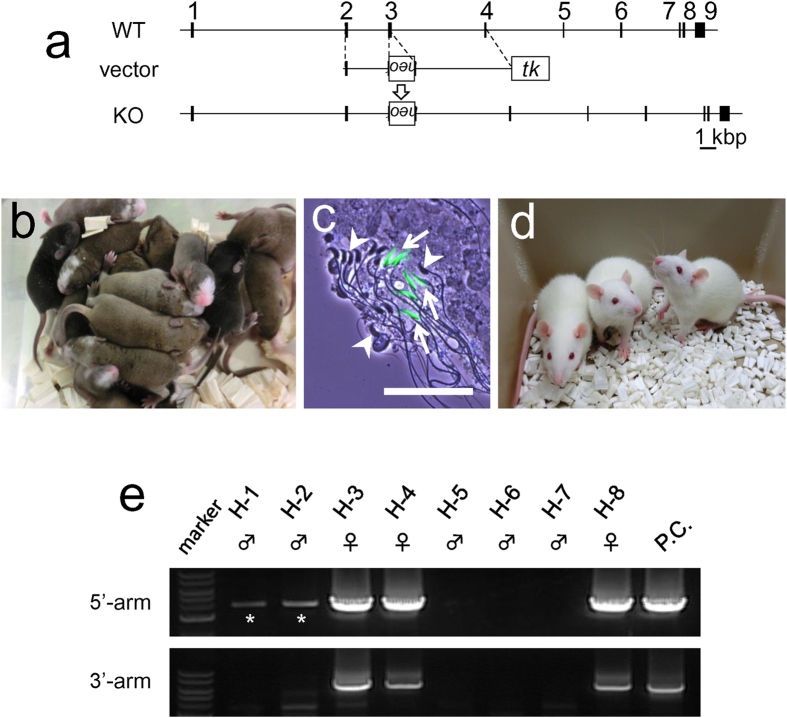
Mouse←rat ES chimera formation after HPRT-targeted mutation. (**a**) Targeting strategy for rat *Hprt* mutation. (**b**) 10-day-old mouse←rat ES chimeras using *Hprt*-targeted GBGS-rat ES cell (rHPRT#5). White coat colour indicates the contribution of ES cells in the chimera. (**c**) *Hprt*-targeted rat spermatozoa indicated by a GFP signal (arrows) and mouse spermatozoa (arrowheads) in the mouse←rat ES chimera. The scale bar shows 50 μm. (**d**) Three *Hprt*-homozygous female rats obtained by TESE-ICSI using spermatozoa from chimeric testis. (**e**) The *Hprt*-targeted allele was identified by PCR. All of the female rats had *Hprt*-targeted allele. As the *Hprt* gene is on X-chromosome, the targeted allele was only inherited by female offspring. Asterisks indicate unpredicted bands.

Since the rat spermatozoa from the chimeric testes were shown to have an ability to produce normal pups, we applied this method to establish a gene-disrupted rat line (Supplementary Fig. S2). We disrupted X-chromosome linked *Hprt* from ES cell lines in the conventional manner (Fig. 4a). After G418 and GANC selection, the cells were further treated with 6-thioguanine to eliminate the wild type cells with HPRT activity. We obtained three *Hprt*-targeted clones with normal karyotypes in more than 70% of the cells (Supplementary Table S3).

Mouse←rat ES chimeras were produced using three ES cell lines (Fig. 4b) and we found rat spermatozoa in chimeric testes from one *Hprt*-targeted clone (Fig. 4c, and Supplementary Table S4). The offspring were generated by TESE-ICSI from these spermatozoa (Fig. 4d) and the disruption of *Hprt* gene in the pups was confirmed by PCR genotyping (Fig. 4e and Supplementary Table S2). As expected, all of the female pups had the *Hprt*-KO allele, whereas none of the males had it.

### Phenotype analysis of *Hprt*-disrupted rats

**Figure 5:**
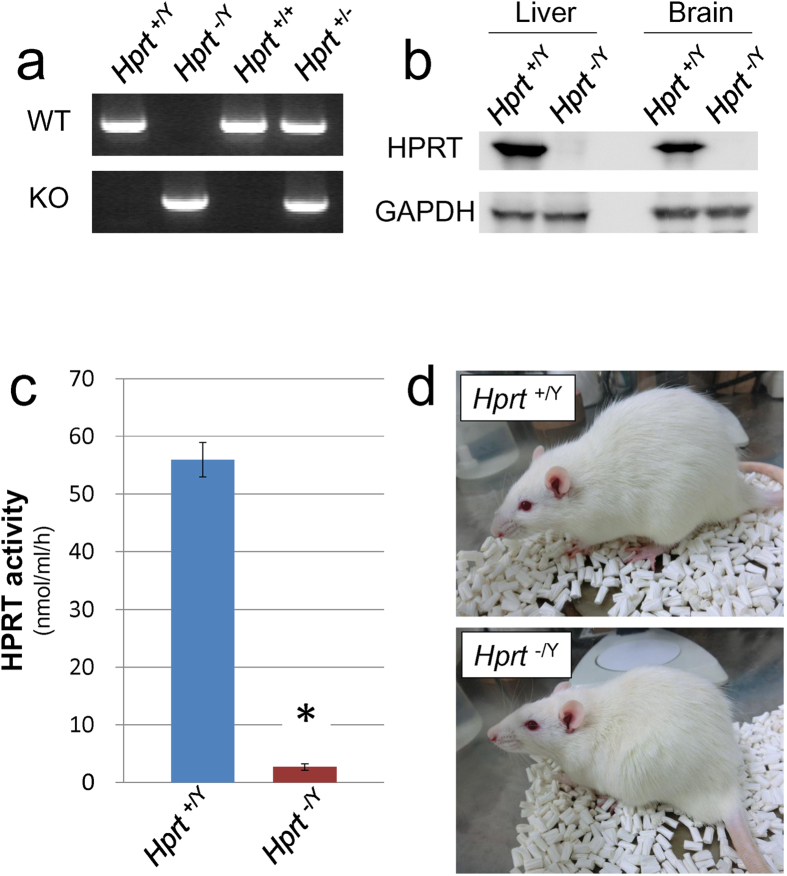
Generation of *Hprt-*disrupted rat. (**a**) Genotyping for the detection of the *Hprt*-targeted allele. (**b**) Detection of HPRT protein in liver and brain using anti-HPRT1 antibody (15059-1AP, proteintech, 1:2000) by Western blot analysis. (**c**) HPRT activities in the brain extract were measured by a PRECICE^®^ HPRT Assay Kit (#K0709-001-2). Three male rats were used in each group. Asterisk represents significant difference (P < 0.01, Student t-tests). (**d**) *Hprt*-deficient adult male rats. The upper panel shows *Hprt*^+/*Y*^ rat, while the bottom panel shows *Hprt*^−/*Y*^ rat. Self-inflicted scratches were not observed in *Hprt*^−/*Y*^ and *Hprt*^+/*Y*^ male rats.

The *Hprt*-heterozygous mutant female rats were mated with F344 male rats and offspring were genotyped by PCR (Fig. 5a). As a result, we obtained 26 (22%) *Hprt*^+/*Y*^ and 21 (18%) *Hprt*^−/*Y*^ males, as well as 37 (32%) *Hprt*^+/+^ and 32 (28%) *Hprt*^+/−^ females, indicating targeted *Hprt* allele was inherited at a normal Mendelian frequency.

The disappearance of HPRT protein in liver and brain, and the demise of HPRT activity (*Hprt*^+/*Y*^ 55.9 ± 3.0, vs. *Hprt*^−/*Y*^, 2.7 ± 0.6 nmol/ml/h; *P* < 0.01) in the extract of brain homogenate were shown in *Hprt*^−/*Y*^ rats (Fig. 5b,c). The *Hprt*^−/*Y*^ rats were maintained for more than a year, but they showed healthy, with no signs of abnormality in their behaviour, e.g. SIB-like behaviours (Fig. 5d).

## Discussion

Lesch-Nyhan syndrome (LNS) is known to be caused by a mutation of the *HPRT* gene leading to a deficiency or complete absence of HPRT enzyme activity. LNS patients suffer from an overproduction of uric acid which may lead to the development of uric acid crystals or stones in the kidneys, ureters, or bladder. Disorders of the nervous system and behavioural problems such as self-injury behaviour (SIB) are also common symptoms. *Hprt*-deficient mice have been produced to establish an animal model for this syndrome, but these animals exhibit none of the neurobehavioral abnormalities including SIB^18^. One could argue that the presence of uricase in rodents could be the cause of this difference, but the occurrence of SIB has not been affected in LNS patients when the uric acid level is lowered by xanthine oxidase inhibitors^19^.

Studies of human brains have suggested that the neurological symptoms of LNS could be related to dysfunction of the dopaminergic neurotransmitter system^20^,^21^. Although, the *Hprt*-disrupted mice showed lowered dopamine levels in the brain, SIB-like behaviour was not observed^22^. On the other hand, a forced reduction of dopamine with 6-hydroxydopamine in rats during their development is used as a model of the dopamine deficiency in LNS^23^.

Combining these facts together, we produced *Hprt-*disrupted rat, exploring possible production of an animal model for LNS. However, the *Hprt-*disrupted rats did not show any SIB-like behaviour or detrimental effect on health in the environment of our animal facilities. The role of *Hprt* gene in rodents (at least in mice and rats) seemed to be less essential than in human.

It is known that rat spermatogonia injected into mouse testes are supported by mouse Sertoli cells and undergo spermatogenesis to produce rat spermatozoa^24^. The fertilizing ability of these spermatozoa was proven by producing pups by microinsemination^25^. A new gene disruption method using rat germline stem (GS) cells has been explored using this xenogeneic transplantation system. Homologous recombination was successfully demonstrated in rat GS cells, but no offspring were obtained using rat spermatozoa from mouse testis even though the original rat GS cells produced fertile spermatozoa^26^. As the researchers also speculated, the karyotype of rat cells in culture, especially when exposed to a low serum concentration, is prone to develop abnormalities. In case of the rat spermatozoa derived from mouse←rat chimeric testes, it was reported that rat iPS cell-derived spermatozoa yielded offspring^27^. However, fertilizing ability of spermatozoa after homologous recombination was not examined. This is the first report that homologous recombined rat spermatozoa could be generated in the mouse←rat ES chimeric testis.

We previously reported that using a *Acr3-EGFP* transgene enabled observation of accumulated EGFP in sperm acrosome^17^. However, Tsukiyama *et al*. reported that EGFP was not detectable in rat spermatozoa derived from the mouse←rat iPSC chimeric testis using *CAG*/*Acr3-EGFP*^27^. In our experiment, we established ES cell lines from *CAG*/*Acr3-EGFP* double transgenic rats confirmed to have green fluorescent in acrosome. This enabled us to identify and retrieve rat spermatozoa despite their small number among countless mouse spermatozoa in the chimeric testis.

The phenotypes of human disease can sometimes be reproduced in KO rat more resembled manner than in KO mouse^28^,^29^. Therefore, if establishing a human disease animal model in mouse is difficult, it merits a try in rat. Nevertheless, reports of KO rat using ES cells through homologous recombination remain few^6^,^7^,^8^,^9^,^10^.

The reason for this has not been analysed sufficiently, but our experience and previous reports^5^,^6^,^26^ suggest that it could be attributable to low GLT ratio derived from fragile characteristics of rat ES cells *in vitro* cultivation. The lower the GLT ratio, the more mating pairs are required. It may also require longer breeding periods, labour, and cost. Altogether these challenge production of gene-disrupted rat. One advantage of our mouse←rat ES chimera system is simple, early-stage identification of GLT by direct observation of rat spermatozoa in whole testis occurring as early as 10 weeks of age. Another advantage is that required animal room space almost equals that of mouse KO experiments.

Recent, genome editing technologies such as ZFNs, TALENs, and the CRISPR/Cas system function as powerful tools in production of gene modified animals^30^,^31^. However, for complicated gene modifications such as knock-in, conditional knockout, gene trap and chromosome engineering, ES cells may retain advantages in large-scale experiments to detect rather rare events.

Our chimeric testis method described here provides a new practical method to produce a gene-manipulated rat.

## Methods

### Animals

All animal experiments were conducted in accordance with the guidelines of “Animal experiment rules” established by the Research Institute for Microbial Diseases, Osaka University, and were approved by the Animal Care and Use Committee of the Research Institute for Microbial Diseases, Osaka University. F344, and SD rats, ICR and BDF1 mice were purchased from Japan SLC, Inc. Severe combined immunodeficiency (SCID) mice (BALB/c JHan Hsd-Prkdc scid.) were purchased from Clea Japan, Inc. Wistar rats were purchased from CHARLES RIVER LABORATORIES JAPAN, INC.

The double transgenic rat lines were produced by injecting a mixture of two transgenes (*CAG-EGFP* and *Acr3-EGFP*)^16^,^17^ into the pronucleus of F344 fertilised eggs.

### Construction of the targeting vector

The rat *Hprt* gene consists of nine exons and the targeting vector was designed to remove a part of the third exon of *Hprt* (Fig. 4a). A targeting vector was constructed using pNT1.1 [ http://www.ncbi.nlm.nih.gov/nuccore/JN935771]^32^. For *Hprt* gene disruption, a 2.7 kb NotI-XhoI fragment as a short arm and a 5.7 kb KpnI-XbaI fragment as a long arm were obtained by PCR amplification using genomic DNA derived from F344 as a template. The PCR primers used were (5′-3′): AAGCGGCCGCATTAGTGATGATGAACCAGGTTATGACC and TTCATGACATCTCGAGCAAGTCTTTCAGTC for the short arm (SA) and AAGGTACCTGTAGATTTTATCAGACTGAAGAGCTACTG and CTTTCCAGTTAAAGTTGAGAGATCATCTCC for the long arm (LA). DNA fragments were amplified using KOD FX (TOYOBO) for 40 cycles under the following conditions: 94 °C for 30 sec, 68 °C for 3 min (SA) or 8 min (LA). These two fragments were inserted into a pNT1.1 vector and the targeting construct was linearized with NotI.

### Generation of *Hprt*-disrupted ES cells and genotyping

Rat ES cells were established and maintained in 2i containing medium as described^12^. For homologous recombination, 1.6 × 10^7^ rat ES cells were electroporated with 50 μg of linearized DNA in a total volume of 800 μl using a Bio-Rad Gene Pulser (250 V and 500 lF; Bio-Rad, Foster City, CA, USA). They were then plated onto 1% matrigel coated 100-mm tissue culture dishes plated with SNL cells in N2B27-2i medium. The rat ES cells were treated with 75 μg/ml of G418 at two days and 4 μM of GANC at four days after electroporation for positive/negative selection. Ten days after electroporation, 284 survival colonies were retrieved and cultured with 10 μM 6-thioguanine for obtaining of *Hprt-*disrupted clone (17 clones). The correct targeting was confirmed by PCR analysis for homologous recombination on both the 3′ and 5′ ends of the targeting vector.

Screening primers for ES cell clones were (5′-3′): GTATGCTGGGCTCTGGACATAC and GCCTTCTATCGCCTTCTTGACGAGTTCTTC for the short arm and CCGGTGGATGTGGAATGTGTGCGAGGCC and CTTTCCAGTTAAAGTTGAGAGATCATCTCC for the long arms. DNA fragments were amplified using Ex Taq (Takara) and Ampdirect (SHIMADZU) for 40 cycles under the following conditions: 94 °C for 30 sec, 68 °C for 3 min (SA) or 8 min (LA).

Genotyping primers for the detection of *Hprt-*disrupted rat were (5′-3′): TAGTCCAGGCTGTCTTTCAACTC (rHPRT-F1 for typing), GCTTTAATGTAATCCAGCAGGTCA (rHPRT-R1 for typing) and ATCTGGACGAAGAGCATCAG (Neo-Pr114 for typing). DNA fragments were amplified using KOD FX (TOYOBO) for 40 cycles under the following conditions: 94 °C for 30 sec, 65 °C for 30 sec and 72 °C for 1 min.

### Production of the mouse←rat ES chimera

The mouse←rat ES chimeras were produced as described^12^. Briefly, Two- or four-cell stage embryos were collected from E1.5 pregnant mice which were BDF1 females crossbred with ICR males. After incubation for 48 hours, four to six established rat ES cells were injected into each mouse blastocyst. Subsequently, mouse←rat ES chimeric blastocysts were transferred into the uterine horn of E2.5 pseudopregnant ICR mice. Mouse←rat ES chimeras were recovered by natural delivery on E19.5 or by Caesarean section on E19.5, and were identified by GFP fluorescence and coat colour.

### Microinsemination

For TESE-ICSI, male mouse←rat ES chimeric testes were obtained from animals aged ten weeks or more. After removing the tunica albuginea, seminiferous tubule fragments showing EGFP signal in the mouse←rat ES chimera were carefully dissected using fine forceps under the fluorescence microscope. GFP-positive spermatozoa extruded from the isolated tubules were suspended in Hepes-mR1ECM medium. Their unique head shapes identified rat spermatozoa. Rat spermatozoa were subsequently microinjected into oocytes as described previously^14^. On the same day, constructed eggs were transferred into the oviductal ampulla of E0.5 pseudopregnant SD rats. Offspring were recovered by natural delivery on E21.5 to E22.5 or by Caesarean section on E22.5.

## Additional Information

**How to cite this article**: Isotani, A. *et al*. Generation of *Hprt*-disrupted rat through mouse←rat ES chimeras. *Sci. Rep.* **6**, 24215; doi: 10.1038/srep24215 (2016).

## Supplementary Material

Supplementary Information
