# Supplementary material for: Generation of Hprt-disrupted rat through mouse←rat ES chimeras
Source: Sci Rep. 2016 Apr 11;6:24215. doi: 10.1038/srep24215 (PMC4827079; doi:10.1038/srep24215)
Supplement: Supplementary Information [file srep24215-s1.pdf]

Supplementary Information

Generation of *Hprt*-disrupted rat through mouse←rat ES chimeras

Ayako Isotani[1,2,*], Kazuo Yamagata[2,3], Masaru Okabe[2] and Masahito Ikawa[1,2]

1. Immunology Frontier Research Center, Osaka University, Yamadaoka 3-1, Suita, Osaka 565-0871, Japan

2. Research Institute for Microbial Diseases, Osaka University, Yamadaoka 3-1, Suita, Osaka 565-0871, Japan

3. Present address: Department of Genetic Engineering, Faculty of Biology-Oriented Science and Technology, Kinki University, 930 Nishimitani, Kinokawa, Wakayama 649-6493, Japan

* Corresponding author: isotani@biken.osaka-u.ac.jp

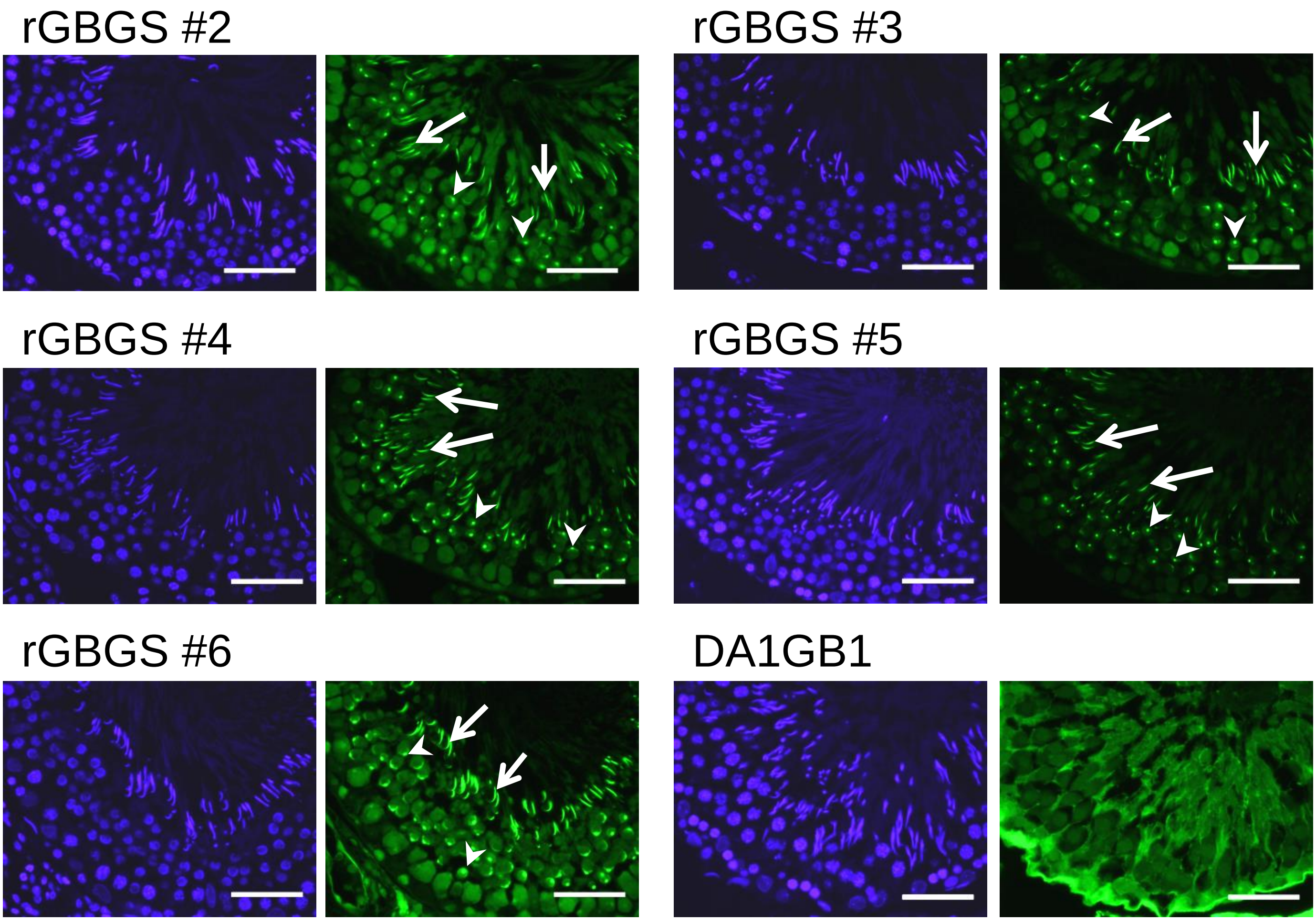
rGBGS #2
rGBGS #3
rGBGS #4
rGBGS #5
rGBGS #6
DA1GB1

Supplementary Figure S1 Testicular section of GS-rat and GBGS-rat

Among six transgenic founder rat lines we produced (rGBGS#1 to rGBGS#6), the rGBGS#1 was infertile. The testicular sections of the remaining 5 transgenic rat lines established are shown in the figure (rGBGS #2 to #6). The blue indicates Hoechst staining, while the green indicates the fluorescent GFP. The testicular section of our previously-reported transgenic rat line DA1GB1, which has no green acrosome, is also shown in the panel. Note that all GBGS-rat lines had acrosomal GFP shown as bright dots (arrowheads) and bright falcate dots (arrows) in the figures. Arrowheads indicate round spermatids, while arrows indicate elongated spermatids. Scale bars indicate 50 μm.

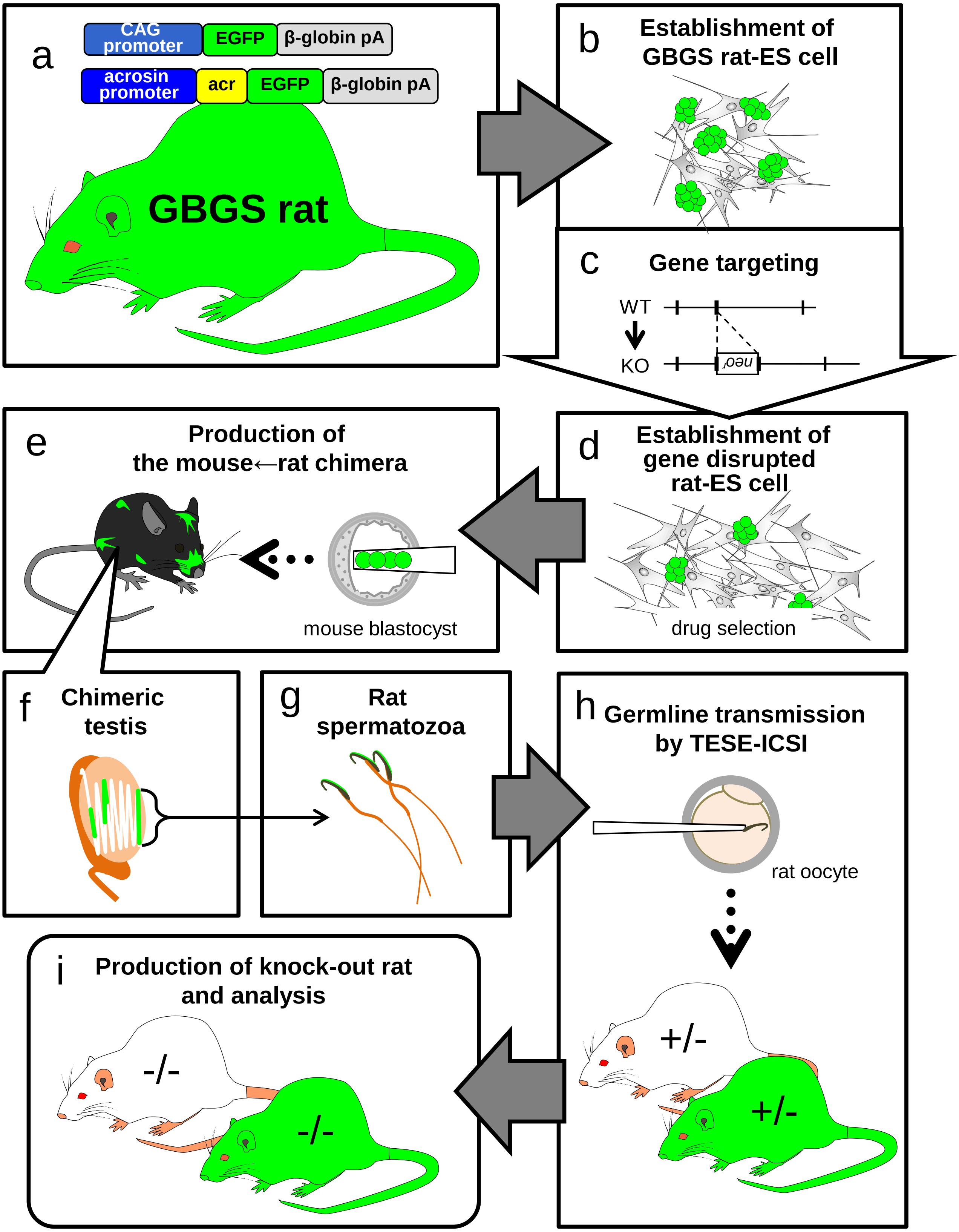
a
CAG promoter
EGFP
β-globin pA
acrosin promoter
acr
EGFP
β-globin pA
GBGS rat
b
Establishment of GBGS rat-ES cell
c
Gene targeting
WT
KO
neo
d
Establishment of gene disrupted rat-ES cell
drug selection
e
Production of the mouse←rat chimera
mouse blastocyst
f
Chimeric testis
g
Rat spermatozoa
h
Germline transmission by TESE-ICSI
rat oocyte
+/-
+/-
i
Production of knock-out rat and analysis
-/-
-/-

Supplementary Figure S2 Schematic diagram of the establishment of a knockout rat line through the production of mouse←rat ES chimera

(a, b) The rat ES cells were prepared from double transgenic GBGS rat line, which harbours double transgene *CAG-EGFP* and *Acr3-EGFP*. This transgenic rat line had ubiquitous green fluorescence in both body and sperm acrosome (The coat colour was not green, but the rat is shown as green in the figure for convenience). (c, d) The gene-disrupted rat ES cell lines were established by homologous recombination. (e) The rat ES line was injected into mouse blastocyst following the homologous recombination in the conventional manner and the chimeric embryos were transplanted to mouse recipient mothers. (f, g) Germ line transmission was determined by identifying rat spermatozoa in the chimeric testes. Since the GFP was loaded in acrosome from the chimeric mouse testes, rat spermatozoa were easily identified by the green fluorescence. (h, i) The isolated rat spermatozoa were injected into unfertilised rat eggs and transplanted into pseudopregnant rat mothers. Knockout rats were subsequently established by mating and genotyping.

Supplementary Table S1

Generation of mouse←rat chimera using rat GBGS- ESC lines

type of ES cell	line ID	No. of passage	No. of chimeric embryos transffered	No. of pups	No. of weaned chimera	
					♂	♀
F344-GBGS	05	16	80	13	0	0
	07	8	60	24	7*	3
	08	8	60	15	2*	4
	total		200	52	9	7
WI/F344-GBGS	101	9	40	11	1*	2
	102	9	40	13	2**	1
	104	9	78	27	3**	3
	104	16	80	31	3**	3
	total		238	82	9	9

*: Rat spermatozoa were not found in these males.
**: One male had rat spermatozoa in chimeric testes, respectively.

Supplementary Table S2

Generation of rat from the mouse←rat chimera using TESE-ICSI

ES cell line of origin for sperm	No. of oocytes injected	No. of embryos transferred (%)	No. of pups (% ET)
rGBGS-ES-104	121	93 (76.9%)	2 (2.2%)
rHPRT#5	447	396 (88.6%)	10 (2.5%)
total	568	489 (86.1%)	12 (2.5%)

Supplementary Table S3

Karyotype analysis of the *Hprt*-deficient rat GBGS-ES cell lines

clone ID	No. of passage	Frequency of karyotype (No. of chromosome)		
		(< 42)	(42)	(42 >)
rHPRT #2	13	18%	73%	9%
rHPRT #3	13	60%	40%	0%
rHPRT #4	13	30%	30%	40%
rHPRT #5	13	10%	90%	0%
rHPRT #6	13	80%	20%	0%
rHPRT #7	13	80%	20%	0%
rHPRT #8	13	50%	40%	10%
rHPRT #9	13	60%	0%	40%
rHPRT #10	13	36%	64%	0%
rHPRT #11	13	50%	30%	20%
rHPRT #12	13	30%	70%	0%
rHPRT #13	13	70%	30%	0%
rHPRT #14	13	0%	20%	80%
rHPRT #15	13	40%	60%	0%

ES cells of clone ID rHPRT #1, #16 and #17 could not promote outgrowth after replating, respectively.

Supplementary Table S4

Generation of mouse -rat chimera
using the *Hprt*-deficient rat GBGS-ES cell lines

clone ID	No. of passage	No. of chimeric embryos transffered	No.of pups	No. of weaned chimera	
				♂	♀
rHPRT #2	13	60	17	3*	7
rHPRT #5	13	67	28	12**	10
rHPRT #12	13	60	23	0	0

*: Rat spermatozoa were not found in these males.
**: four out of 12 males had rat spermatozoa in chimeric testes.
